# A day in the life: psychological impact on emergency responders during the 22 March 2016 terrorist attacks

**DOI:** 10.3389/fpsyt.2024.1353130

**Published:** 2024-02-12

**Authors:** Emilie Muysewinkel, Lara Vesentini, Helena Van Deynse, Stephanie Vanclooster, Johan Bilsen, Roel Van Overmeire

**Affiliations:** ^1^ Mental Health and Wellbeing Research Group, Vrije Universiteit Brussel, Brussel, Belgium; ^2^ Department of Public Health, Vrije Universiteit Brussel, Brussel, Belgium

**Keywords:** terrorism, emergency responders, stressors, debriefings, mental health

## Abstract

**Introduction:**

Terrorist attacks can cause severe long-term mental health issues that need treatment. However, in the case of emergency responders, research is often vague on the type of stressors that emergency responders encounter. For example, in addition to the threat that they work under, studies have shown that ill-preparation adds to the stress experienced by emergency responders. However, few studies have looked into the experience of emergency responders. In this study, we looked at the experience of emergency responders during the 22 March 2016 terrorist attacks in Belgium.

**Methods:**

We used a qualitative design, in which we interviewed different types of emergency responders. Police officers, nurses, soldiers, firefighters, and Red Cross volunteers were included. Interviews were coded by two researchers and analyzed using a thematic approach.

**Results:**

Four large themes were developed: constant threat and chaos, frustrations with lack of preparedness and training, ethical decisions, and debriefings. In addition, although emergency responders encountered constant threat, they often felt that they were ill-prepared for such attacks. One specific example was their lack of training in tourniquet usage. Furthermore, in a disaster setting, the emergency responders had to make life-and-death decisions for which they were not always prepared. Finally, debriefings were conducted in the aftermath of the attacks. Whereas most were perceived as positive, the debriefings among police officers were viewed as insufficient.

**Conclusions:**

Emergency responding to terrorist attacks has many different dimensions of events that can cause stress. Our study revealed that preparation is key, not only in terms of material but also in terms of ethics and debriefings.

## Introduction

Terrorist attacks can cause severe mental health reactions among emergency responders. Although, overall, emergency responders have fewer reactions than the general population (1, 2), studies after 9/11 have shown that, in both the short term and the long term, mental health disorders such as post-traumatic stress disorder (PTSD) and depression can occur after exposure to terrorist attacks (2). Studies after the attacks in France, Norway, or Germany showed similar results (1, 3–8).

Few studies shed light on the many different stressors that emergency responders experience. Furthermore, there is little insight in how proper preparation for such events plays a role, how the feeling of “knowing what to do” acts as resilience against stress, or how having the right equipment can increase or decrease the sense of threat and sense. However, studies have shown the importance of such elements in how stress is experienced during the acute phase of a terrorist attack (9, 10). For example, depending on the type of disaster, different preparations will be necessary (11). Therefore, it is expected that, in addition to the terrorist attack itself, other factors will play a role in acute stress, but also future possible PTSD or depression (11).

A reason for a lack of studies is that most terrorism studies have concentrated on measuring PTSD (12, 13). However, studies on emergency responders have shown the importance of looking at how preparation and response relate to psychological responses. For example, one study showed that hospital personnel after the Manchester Attacks in the United Kingdom felt stress due to being ill-prepared for the massive inflow of victims and felt guilty for not being able to work at the scene of the terrorist attacks (9). Another qualitative study showed that stressors are also the scene in which emergency responders work, as each large disaster is, in a way, unique (10, 11).

Furthermore, few studies have looked into the immediate post-action debriefing. Despite the importance of early psychosocial aid, few studies have looked at how such post-action aid is provided (14, 15). However, here, too, more insight is needed. For example, emergency responders’ culture might play a role, with some not wanting to show emotions, due to something that some authors have called “John Wayne syndrome” (named after the famous Hollywood actor who often played cowboys) (10). Furthermore, to ensure that the guidelines on post-response aid are improved, more studies on the experiences and needs of emergency responders in the acute phase of an attack are necessary (9, 10, 15, 16).

In short, we have little insight into what emergency responders are exposed to in terms of stressors during their actions after a terrorist attacks and how they are aided in the immediate aftermath. Studies can help in explaining the multitude of issues and stressors emergency responders face during their actions, as well as what aid might be appropriate in the aftermath of terrorism (9). Furthermore, without studies on the psychological impact on emergency responders during the acute phase of a terrorist attack, it becomes impossible to improve both the preparedness and the response of emergency responders in terms of their resilience.

Thus, in this study, the experience of emergency responders during the acute phase and post-action aid of a terrorist attack were studied. The terrorist attack that was examined was the 22 March 2016 attack in Belgium.

### The terrorist attacks of 22 March 2016

On 22 March 2016, 32 innocent people were killed in several bombings, with around 340 people injured (14). First, two bombs went off at the national airport. These attacks were followed by a bombing in a metro station in Brussels. All over Belgium, emergency responders had to help victims of the attacks in a situation of constant threat, both at the place of the scene and at the hospitals (17).

## Methods

### Design

The broad aim of the study was to gain insight in the experiences of emergency responders who have to respond to a disaster such as a terrorist attack. We particularly wished to find 1) what stressors emergency responders experienced and their psychological impact and to identify 2) factors that aided or limited their response and 3) their experience of the post-action debriefings. To achieve this, a qualitative design was employed, and in-depth interviews were conducted.

### Participants

We interviewed emergency responders who were put into action at the attacks on the national airport.

The emergency responder groups that were included were as follows:

- Military personnel
- Nurses
- Police officers
- Firefighters
- Red Cross volunteers

Emergency responders could be both directly and indirectly exposed. Directly exposed was seen as being present at the terrorist attacks in function of their work. Indirectly exposed was seen as not being present at the scene of the terrorist attacks, but coming into contact with victims of the attacks (e.g., a nurse in a hospital). Military personnel were included because, at the time of the attacks, they provided security for the airport and, thus, were the first responders.

Exclusion criteria were as follows:

- Respondent was not an emergency responder (e.g., airport employee or traveler).
- Respondent did not speak Dutch or English.
- Respondent was involved in the response at the metro station and not at the national airport.

We focused in this study on the national airport, due to several reasons. First, there were more emergency responders involved in the direct response, because 1) it preceded the metro attacks (and thus more emergency responders across the country responded) and 2) it was a larger attack in terms of scale (although, the number of fatalities was similar). Second, to assess the experience of the emergency responders, it was thought to be more opportune to focus on one of the two events. The airport was an attack in a large area, where there were possibilities for future attacks, whereas the attack in the metro was a close quarter attack. Thus, in terms of understanding the stressors faced by the responders, it seemed important to focus on one attack. Third, as the airport is in Flanders, Belgium (where the main language is Dutch), it was easier for the researchers to find enough respondents who spoke Dutch, whereas the metro station is situated in Brussels, where French is the dominant language.

In total, 29 emergency responders were included, of which five were indirectly exposed (see **Table 1**). Of these, nine later sought out professional mental health aid, of which seven were part of the police force. The division in terms of gender was 26 men and three women. All participants were white and Belgian natives. The years of experiences were diverse, although the majority had more than five years of experience.

**Table 1:** Characteristics of respondents.

	Emergency occupation	Age	Gender	Exposure	Sought out professional mental health aid later on?
1	Police	50–60	Male	Direct	Yes
2	Police	50–60	Male	Direct	Yes
3	Police	50–60	Male	Direct	Yes
4	Police	40–50	Male	Direct	Yes
5	Police	40–50	Female	Direct	Yes
6	Police	40–50	Female	Direct	Yes
7	Police	50–60	Male	Direct	Yes
8	Police	50–60	Male	Direct	No
9	Police	40–50	Male	Direct	No
10	Police	40–50	Male	Direct	No
11	Red Cross volunteer	50–60	Male	Indirect	No
12	Red Cross volunteer	30–40	Male	Indirect	No
13	Red Cross volunteer	20–30	Male	Direct	No
14	Soldier	20–30	Female	Direct	Yes
15	Soldier	20–30	Male	Direct	Yes
16	Soldier	20–30	Male	Direct	No
17	Soldier	20–30	Male	Direct	No
18	Firefighter	40–50	Male	Direct	No
19	Firefighter	40–50	Male	Direct	No
20	Firefighter	50–60	Male	Direct	No
21	Firefighter	40–50	Male	Direct	No
22	Firefighter	40–50	Male	Direct	No
23	Firefighter	50–60	Male	Direct	No
24	Firefighter	30–40	Male	Direct	No
25	Nurse	40–50	Male	Indirect	No
26	Nurse	60–65	Male	Indirect	No
27	Nurse	30–40	Male	Indirect	No
28	Nurse	50–60	Male	Direct	No
29	Nurse	50–60	Male	Direct	No

### Data collection

A semi-structured topic guide was used (see **Table 2**). The topic guide was developed on the basis of several previous studies, including similar qualitative studies (9, 10), quantitative studies and reviews (14, 18, 19), and studies on debriefings (15, 16). After development, the interview guide was reviewed by the research group, with several researchers having experience in qualitative research.

**Table 2:** Topic guide.

1. Description of hearing of the attacks 2. Role in the response 3. Feeling of preparation 4. Description of the response 5. Issues experienced in response

Information about the study was spread through emergency responder organizations (e.g., sites of fire departments of the region). Afterward, through the snowball techniques, respondents were recruited. Interviews took place between mid-2018 and the beginning of 2019.

Half-structured interviews were conducted. Interviews were always conducted in person, at a place of choice of the respondent. Respondents were asked to ensure that the place of their choosing allowed for interviews without interferences of colleagues. Interviews typically lasted between an hour and an hour and a half. Respondents were always informed prior to the interview of the possible length of the interview.

Data collection continued until data saturation, meaning no new information was found. Because our sample is quite diverse, data collection continued until eventually 29 emergency responders were included. This allowed us to take into account the differences between different groups of emergency responders and differences between indirectly and directly exposed emergency responders.

### Data analysis

Thematic analysis was used to categorize and summarize the data (20). Two researchers analyzed the data independently: E.M. and R.V.O. E.M. has a background in public health research and is a PhD student, whereas R.V.O. is a post-doctoral researcher with expertise in PTSD research. Several steps were taken in analysis. These steps were based on previous research on this topic (in which thematic analysis was also used) (9) as well as other papers using thematic analysis (21, 22).

1. Transcription of the interviews was done by the researchers (e.g., not by software making automatic transcriptions). Following this, analysis was done in several steps. After transcription of the interviews, all interviews were read by the two researchers to familiarize themselves with the data.
2. The transcriptions were then coded. Coding meant, in this case, that the explicit meaning was named in the transcriptions. For example, if a respondent mentioned that he or she feared for the safety of their children, then this might be coded as “fear for safety of loved ones.” In this step, no interpretation was yet made.
3. After coding, differences in codes between the two researchers were discussed. In case of disagreement, coders discussed until an agreement was reached. In most cases, codes were the same. Only for codes that would form the theme “constant threat and chaos,” there were some differences, although mainly semantic ones. These were resolved through discussion.
4. Themes were generated. Themes in our study are topic summaries, made up of the codes generated in step 2.
5. Once the themes were generated, these themes were discussed with the other researchers until agreement was reached.

Analyses were conducted using NVivo 12.0. As interviews were conducted in Dutch, translation of quotes was performed by one researcher and then checked for meaning by the other researcher. No artificial intelligence software was used in any step of the study.

### Ethics

All participants were informed about the study, their rights, and potential support channels. Subsequently, they provided their written consent to participate in the study. Information pertaining to the participants’ identities was expunged from transcripts to the fullest extent possible (e.g., names and addresses). All encoded transcripts were securely stored on a server owned by Vrije Universiteit Brussel (VUB), with which all authors are affiliated. Furthermore, this study received approval from the Commission Medical Ethics of UZ Brussels/VUB (B.U.N. 1430201836125).

To ensure anonymity, age was presented in categories.

## Results

Data analyses generated four large themes: constant threat and chaos, frustrations with lack of preparedness and training, ethical decisions, and debriefings.

### Constant threat and chaos

During the acute phase, the main stressor was the feeling that, at any moment, other attacks might occur, causing a sense of constant threat for emergency responders to work in.

“So, we get there. And we were going to discuss: okay, what are we going to do here? And right when we want to begin, we got a message: ‘Everybody get out, get out, there are shooters on the roof.’ So, you send the message around: ‘Boys, get out!’ So, we started running and running and at a certain moment you come to the edge there, and you have the choice: do I jump six meters down? [ … ] You know, we’re not used to that, running away from danger. In our normal interventions, we run towards the danger.” (Firefighter, #23)

“There was supposedly a magazine of a gun found on the other side of A-terminal (at the airport). And then you fear two things: things are going to explode here and then … You don’t have to be a genius to know that: in a moment they’re going to start shooting. Now, apparently the magazine had fallen out a military weapon—it was from a soldier, fell out of his bag. The magazine was atypical for an enemy weapon. But you know, you focus on that for that time, and only then you can go back to the victims. It was so chaotic, but everyone does their part in that chaos.” (Police, #1)

“We went to look for possible shooters—my idea was genre Bataclan (where people inside a concert hall were shot at by terrorist attacks after earlier bombing in the city), that there would come an active shooter. A third bomb, maybe, but mainly an active shooter. So, you know, you had to be alert. But in the meantime, everything came falling down. Like, parts of the ceiling. Dust, chaos, guts of people spread here and there. People just sitting on the floor. Super relaxed. People running around hysterically. People taking out their cell phones who start to film people are bleeding out … I don’t get that. But you know, it was an odd situation (laughs).” (Soldier, #15)

Most of the respondents stated that they reacted to this feeling of threat with fear and stress. It should be noted that some indicated that they had other reactions that participants stated were odd. For example, two participants admitted laughing at morbid scenes, in a way almost to break the tension of the constant threat.

“I laughed that day with things. I was standing there with someone. So, someone was on the ground. There wasn’t a lot of light. And all those hairs were burned. And I was looking and thinking: is that a man or woman? And so I look at their feet. One of them was still hanging on barely, the other one was blown off. And the nails were painted. I mean, lacquered. So, I thought, it’s a woman. And I laughed. I mean, I guess it shows that I really was present at the moment. Because right after that, I thought, okay, I am at the back of the stretcher. So, I have to put that feet up on the stretcher (laughs). Everyone didn’t want to put body parts on the stretchers, because it was cold, dead, papery. Like, if you had to do that, tough luck.” (Police, #5)

“You know, I found that blown up terrorist there. And at the moment, I found that pretty funny. A piece of meat, it really was. And his skull broken open. And his jaws with his little teeth out there. I said ‘Here he is!’ (laughs) I taught that was funny at the time. ‘Asshole.’ You know, when you have foreign missions, and you come to a market place when they slaughter animals—like now, a couple of years ago in Africa, I was walking down the street, and there is a donkey there, and a truck passes and (makes a splashing sound). That donkey imploded.” (Soldier, #15)

For others, the reaction to the threat of others caused a sense of unsafety for them.

“So, at a certain moment, you see police and firefighters running, and you hear them yelling ‘They’re shooting’ or ‘They found a bomb!’ And everyone ran and ran. Very unsettling. Most of them ran away, only a few stayed. But oh boy, I’ll never forget. Outside, on the parking, there was this girl from (Flight agency). Her legs full of holes. And she was hyperventilating, of course. So, you see everyone running away, and I stayed with her. A colleague of mine had jumped behind a truck, and five others on him (laughs). That was unsafer than … You know, they say collective panic isn’t real. But, I can assure you, in that moment, it was. Those police officers who were supposed to protect us, they were running away, you know. So, what to do you then?” (Red Cross volunteer, #11)

Similarly, participants had to deal with the emotional shock of others.

“Right inside the airport terminal (where the attack was). Two dumb bitches from Antwerp. Barbie and Barbie of the third age. With their Antwerp accent. (imitates accent): ‘Ehm, madam, we have to get to Alicante.’ I was like ‘I don’t think that flight will leave right now.’ ‘What do you mean we won’t go?’ ‘I don’t think you realize, but there has been an attack here. There is no airplane taking off.’ So, I say ‘Either you stay here and put your own life at danger, or you make sure you get out of here, so, get the fuck out here.’ So, yeah, those two were in shock.” (Police, #6)

“There were people who were send outside, and that ran back to me and said ‘I have to get inside, inside, my flight is leaving.’ And there were those that were angry and said stuff like ‘You made me drop my newspaper. You are going to buy me a new newspaper.’” (Soldier, #17)

For some, the constant threats toward them were less of a concern than what such threat alarms implied for their loved ones.

“You think about your own … (children) They’re in school, sure, not even close to the attacks, so I don’t think a terrorist would be interested in that, but whatever. You’re thinking about it. So, when you see your children again at home, that’s pretty intense, emotionally.” (Firefighter, #18)

### Frustrations with lack of preparedness and training

In addition to the constant threat, frustrations were outed on the lack of preparedness and training sometimes provided additional stress and insecurity of what to do. This was present in several ways: the way in which communication occurred, the lack of equipment, problems in leadership, and organizational issues.

- Communication

First, there were communication issues. Most communication between emergency responders occurred through a system called ASTRID. As described by some emergency responders, the communication system was quite old, but was developed for small-scale regional disasters.

“They say that communication was overloaded, that the ASTRID system was overloaded. Yeah, true, but (such an attack) is not where ASTRID is normally used for. Normally, it’s in your region, on scan modus, and it works perfectly.” (Nurse, #27)

During the attacks, too many different units and emergency responders were working together, which caused severe delays.

“The system is: you push a button, and you are entered into a list. And you’re at the bottom of that list. As soon as that light is red, you wait. And you get higher in that list until it’s your turn. Queuing that’s called. Sometimes that’s four to five second waiting … That’s long. You have to do that in an extreme stress situation. ‘Goddamn, it stays red,’ so you let go of that button and poof, you’re back at the bottom. And why? Too little discipline for radio communication. Too much being said. Pressure, wanting replies too fast. But also curiosity. I don’t think there wasn’t a firefighter post or ambulance post that wasn’t listening to the disaster channels. But that takes a lot of your capacity.” (Nurse, #28).

The solution for this type of communication came in the form of social media. WhatsApp and other social media were being used to communicate faster and more directly with the people who were needed.

- Equipment

Second, equipment issues were common. A common grievance among the present non-military responders was the lack of training for such large disasters. Especially with regard to tourniquets, several emergency responders felt that they were unable to do everything they could do. A tourniquet is a sort of bandage-device that, when applied to a body part, stops the flow of blood to that body part.

“So, we got a course from 9 (am) to 3 (pm) on tourniquets. And those guys said ‘This course is at least 36 hours, but we’re going to give it in 6 to 7.’ And we all did it once, but that action to do that, you have to know that by heart. And if you a soldier doing that, he does that without looking. And perfectly. And when can you do that? If you do it often, and yearly.” (Firefighter, #21)

Another issue with the tourniquets was that it was taught that using them was illegal.

“So, the soldiers had tourniquets, and they saved lives with that. In our ambulance we didn’t have that. If you used that, you were a murderer. So, that didn’t exist. Now we have those, because they saw that it could be useful. [ … ] But, that’s so Belgian, so they make those tourniquet kits, and so you think Domestic Affairs will hand them to you. No, you can buy them. I don’t get that.” (Firefighter, #20).

The lack of training caused severe powerlessness for many of the respondents.

“Until then we weren’t allowed to use tourniquets in the Red Cross. That was taboo. Because they said you caused more damage than helped people. But we saw dismembered legs and body parts. And those people were bleeding out, and so they put like pressure bandages on that, but you know, that has no point. So, yeah, you feel completely powerless. The soldiers saved a lot of lives, you know. Because we were using like scarfs around the legs … Which is totally ineffective and so frustrating that you can’t do anything at all.” (Red Cross volunteer, #11)

However, from the perspective of soldiers, they also reported that they had wanted more material. Compared to other groups, this did not seem to have been an issue for them in terms of feeling powerless in helping others, which seems to be partly due to knowing what their specific task was, such as securing the area.

“People with limbs and feet off, pieces of their skin scraped away and sticking to the floor – no, I had not seen that before. But not my function either. So, we don’t see that every day or weekly. [ … ] The sergeant said ‘You and you, go to there, and you go there.’ So, we went to the farthest part, you know, with the playground and stuff. And we had to leave people who were laying down, we had to leave them to go there. So, some time later, I see your peloton with medical gear and was like, ‘Over there.’ [ … ] So, our aid-man immediately started applying first care. And you know, there was a woman there who was going into shock, with this gaping hole in here lower back. You couldn’t do anything about that. She was just … Bleeding out. You couldn’t help that. A pity. Anyway, we went on. [ … ] Eventually, the firefighters stormed in, with their paramedics. And we gave them our material—you should know, we did not have a lot. It was a small bag, for personnel use. Which is a shame, because if they expected a bombing, I would think: oh, they’ll give more medical gear. Anyway, what I had left, I gave to that paramedic.” (Soldier, #15)

Other material preparation issues were for example a lack of stretchers or ways to provide oxygen. These issues seemed not to be a severe cause of stress, but simply a cumulation of smaller issues in addition to the general sense of threat.

“So, there were ambulances coming and you see that and say ‘There are no stretchers here.’ Because next to the terminal, there was a post of the firefighters. Come on! With an airport, you kind of have to think that a large crash or a large … That there are at least 500 stretchers ready to go. But it took so long. I wonder if we’re ready now. That if a plane goes into the terminal. Or a fire. That … You cannot really comprehend that … So, what I remember is that we got people out with baggage cars. Like, a guy whose leg was gone. There was nothing else to take them out of the airport. So, you improvise. But imagine the stress of not being able to do what you should do.” (Police, #1)

“Are we in war here, or what? You have no time to ask those questions, because medical personnel run to you: ‘There is not enough oxygen.’ Okay. You get messages on your cell phone. ‘Multiple attacks in Brussels.’ That’s the call we got in Brussel, you know. Multiple attacks … So you think we’re at war, you know. And that works on you. That stress level. You’re so stressed out by the situation, but it just goes up and up. Such a weird situation to work in. And you know, in hindsight, nothing else happened. But your stress just keeps increasing.” (Nurse, #28).

For those indirectly exposed, in the hospital and at the Red Cross response, the problem was more of the nature of a lack of knowledge of the disaster plans. Whereas improvisation was key at the site, this was not the case for hospitals.

“You have to aim your personnel towards something. Because a lot of people start acting with reflexes. They do their routine. So, they treat one patient. But that’s not the point. You have to provide in group. So, they like try to do their best, but they kind of start dismantling the system, which can be frustrating.” (Nurse, #26)

However, here indirectly exposed people generally agreed that in a way they were overprepared, even with the slight hiccups of a lack of knowledge of disaster medicine.

“Once our emergency services were empty, once that was organized, we had time left. We were ready for patients, but only a few eventually came. We were literally waiting for the next patient. By our own standards, we could have done more, but we are dependent on the patients coming from the site. So, we organize towards the worst, and we expect the worst. What I mean by that is that we were expecting a rush of patients. If there would be, we would be ready. I think that normally we are registered to take care of 4 very heavily injured people, 4 medium injured and 12 lightly injured. That’s what we can take in terms of inflow per hour, and that’s what we were prepared for … But the people never came.” (Nurse, #25)

- Problems in leadership

Although none of the respondents indicated that the lack of preparation provided a severe breakdown for them, some respondents worked together with others who were unable to cope with the stress of the situation.

“Okay, take X, from the X hospital. He has been head of emergencies; he is now director as well. That guy hasn’t been in an ambulance for 25 years. He knows no one on the terrain. He has no expertise in that. Sure, he’ll have theoretical expertise. But working on the terrain, operational … He can’t do that. I have seen it in smaller disaster, you know, as well. People’s masks fall off; he couldn’t cope with the stress. There has to be a better selection of people who are operational chiefs, who make decisions, and we need to get off of the idea that that has to be an M.D. You need a clear profile. How can you get that expertise? We have no large disasters, but you still need to have this small group, and yes, teach them the theory, but also do a lot of exercises, and a lot more exposure to real situations.” (Nurse, #29)

“You need expertise do exercises. The best example: X, good doctor, nothing to remark on him as a person. French speaking. Knows no one on the terrain. And he panics. I’m telling you, he slipped up. No expertise, no resilience. But, he IS a good M.D. But, just because you’re a good M.D., doesn’t mean you’re a good manager. That’s … That’s difficult. I’m a nurse, and if I’m going to give orders to M.D.’s … That’s difficult, you know.” (Nurse, #28)

Furthermore, another issue in leadership was clearly visible among police forces. It seems that through issues in hierarchy, there were difficulties of putting officers into action.

“Why were so many people there doing nothing? I’m not angry for that, but even then, it were like the first things that made me think … I mean, it started raising questions. Like ‘How can it be that they are standing outside, and we are inside (in the airport) doing this job with just five men. So, why aren’t other officers put into action?’ Yeah, command really failed that day. If they had told those people what to do, then … But it never happened that day. [ … ] But the police is still not prepared to admit they failed that day. I’ll admit, you can’t plan for a terrorist, you’ll never be prepared. But … You know, there are so many incompetent people, but you know, they’re still officers.” (Police, #5)

The issues in leadership in the police forces were noted by other emergency responders as well.

“Nobody of the police showed up in the CP-OPs (coordination of emergency situation). Everyone asked ‘So, is anyone coming?’ Because terror is police. And you know, there weren’t that many fires to put out. And then it turned out that the police—look, there wasn’t a single command. There were five, six commands together. And it was apparently problematic to coordinate. [ … ] So, we were—there was one from the firefighters, with me there to assist, one from public health, and an assistant, and one from civil protection services. But there fifteen from the police. And everyone had to have their say. You could not get a single answer. Chaos! And they all had a lot of gold (refers to medals), so had a lot to say. But that doesn’t work! In the meantime, my colleagues were on their knees on the terrain, saying ‘Send me someone from the police!’ But no one goes. Off they send some asshole who says ‘I have no idea what to do here.’ There was never a single command from the police.” (Firefighter, #21)

Despite threats, equipment problems, and leadership problems, several aspects ensured that care was provided in the field. First, an aspect touched upon by most participants was knowing their colleagues. This was often through going to trainings together or acting together in other disasters.

“When X came along, you think: ‘Oh, I know that guy, he’s resilient, knows his stuff.’ You do that, X, you do that. You need to know each other; it is the key to success. Knowing people on the terrain. Because it provides you with the comfort that you know something will be done right. You really can’t just depend on their uniform. Because, for example, almost everyone will wear of these fluorescent vests. Everyone. Firefighters. Even the garbage men wear that, you know. The system gets completely lost, you get lost, if you don’t have that immediate face recognition. You Need To Know The People!” (Nurse, #28)

However, in this respect, it seems that here there was also a major problem in the cooperation with police.

“Some disciplines … we just can’t get them involved. Medical, sure. Police, nope. They just won’t come. [ … ] I have no idea why. It’s like, we’ll do our thing if we must. But, if it comes to terror, the plans say that some officer of the police will take charge. So, it’s important that that guy comes outside and that you have seen him before.” (Firefighter, #20)

Second, related to the first point was the importance of experience of disaster management. Knowing how to set up a triage system and being able to rely on the tasks other people do ensured that emergency responders were able to simply do their tasks in a system.

### Ethical decisions

Throughout the action, emergency responders had to make several ethical or moral decisions. For example, when there were orders to run away, because there were possible shooters or a bomb car, responders had to make a decision to either leave their patient (leaving them to possibly die) or to stay and possibly die themselves.

Similarly, throughout providing first aid, especially those with a medical training had to decide on life and death for some, as disaster medicine mandates that resources have to go to those that can be saved. This caused not only emotional stress because caregivers could not provide the care they would normally provide but also caused responders to suddenly decide on life and death.

“It’s a simple system: T1, T2, T3, T4. T1: heavily injured, needs aid or is going to die. T2: injured, but can wait. T3: light injury. T4: those are the difficult ones. They require too many resources to help. But … You’re not going to let those people just die. You give comfort therapy. You look at other victims, who are already stabilized. And then you look back at the T4’s. But … That’s such a vague boundary. You just need to decide. And you’re playing God then. And when I teach about it in disaster medicine, I say: ‘That’s the most difficult position to be in.’” (Nurse, #29)

“You can’t concentrate on one person. You can’t organize it like that, it becomes chaos. And you know … More and more people came to us. It was just, so much. [ … ] It’s so difficult to say: who should receive aid first. Who can wait? That’s … Those are difficult choices. You make them on sight, you know. You can’t really investigate. You say: ‘Oh, that one does not look too pale, not too much blood loss, so, that’s a T2.’ But I probably made wrong choices. But, that’s unavoidable, I guess.” (Nurse, #26)

For others, the feeling of guilt came from not being able to perform the care they had wanted to perform because they had to provide care for others or perform other tasks.

“I was kind of disgusted by it. You see someone laying there. His leg off. His right leg, that was. And there was pieces of skin. His bone sticking out … Leg off. His lower leg. And his shoe. But … His skin was blown off. So, I come to him, thinking, what is this? But I was very calm. We’re trained for that. In our training, always stress. Anyway, I started applying first care. Gave the tourniquet, and it was difficult for a while, very exciting, but also suddenly stressful. I checked his bleeding. Controlled him. But, I couldn’t stay with him. Because others needed care. So, there was this person van the border control, and I mean, that boy was stable. What else could I do? I told him, if there is anything, or if he falls asleep, call me. What else could I do? So, I went off, but I felt like it was a mistake. I should have stayed. I still think about that even now. I didn’t finish my job with that boy. But, yeah … I got another task then, which was securing our position. Which was needed. But … Yeah.” (Soldier, #16)

For indirectly exposed emergency responders, this was especially harrowing because, in hindsight, they had resources to spare. Because of a properly executed triage system, patients were divided over as many hospitals as possible, thus avoiding an overload of hospitals. This was a lesson learned from earlier disasters, such as the overload that occurred after the disaster after the football game at the Heysel Stadium in 1985. Furthermore, there was frustration that they weren’t able to go to the site to aid more people.

“At the beginning, everyone was so motivated. Oh, can I help with the phones? Oh, I’ll stay. But stuff like that. But then, you get frustrated. Because even surgeons asked ‘So, when are they coming? I stopped a program for this, you know.’ [ … ] And afterwards, you start to ask yourself questions. We had a few victims who came here by themselves. And then it started, this system with ambulances, and triage, and … I do still remember this Asian boy, who had passed away. But we were saying: ‘Look, this is a disaster setting, we can’t do anything about it, we are going to use our resources for those who are still coming.’ And then afterwards you think ‘If I had known it would be this calm, maybe we could have done more for that kid.’”(Nurse, #27)

“We were saying ‘Come on, bring some more.’ But they didn’t. That was our frustration at the time, because we heard that at the debriefing as well. That was the emotion that prevailed: I wish I could have done more, I wish I could have helped more. Me personally, I wanted to go there, but we weren’t allowed to (because of the main central guiding the emergency services).” (Nurse, #25)

Other ethical concerns were raised with regards to whether they as emergency responders can inform family and friends or not.

“Cell phones. Constantly going off. The cell phones of victims. [ … ] I had those cell phones in my hand. You see the name of someone who is calling them. What the hell do you do with that? Skype Conference Call … What do I do? Luckily some said ‘Do not answer that! Just put it back. Hands off that.’ But it’s so horrible. Those are things I’ll never forget. It’s knowing that someone on the other end of that line is desperately calling, looking for news. And you have the key to their questions. But for several reasons you cannot do it … Whatever, you can’t do it.” (Soldier, #16)

### Debriefings

All emergency responder groups, except one, had some form of debriefings after the attacks.

Most firefighters, nurses, and soldiers viewed the debriefings as necessary, whereas police officers generally felt that the stress teams available were not trained enough. Most of these happened either the night itself or in that week. For a lot of these organizations, it was a structural element of their organization, meaning it was planned to occur after disasters.

“What I really like was that … We had three mandatory group conversations with the psychological cell of Defence. And you kind of discussed the bigger image there. Like, ‘Oh, that guy was there, doing that. I was there around that time. That one there, doing that.’ And then you know everyone was doing their job, doing it well, and all those puzzle pieces come together in one big image. And you can create that image of what everyone was doing.” (Soldier, #15)

“(Debriefings) are quite well organized with the fire department. [ … ] Like, 15 to 20 years ago, you got the FIST team. The Firefighter Stress Team. So, you have these FIST antennas, those are people that are trained to do the first psychological care. And they sometimes go on to the terrain, or wait in the base. Those aren’t psychologists, those are just colleagues. People you know. Their only job is to look for signals, signals that someone might need to talk. [ … ] Afterwards, you get a mail. ‘You can call’ or just reply. And that’s completely external, because at the fire department there is a mentality of distrust towards hierarchy. Like ‘I’m not going to tell that, because they’ll use it against me at a promotion.’” (Firefighter, #18)

“So, that Saturday we organized a general debrief. Only for those present at the site. And you know what you see there? It’s incredible really. Like, people, two years before their pension, crying like children. And why? Like, an example: a child is being taken to a hospital, and that child says ‘Where is my brother? Where is my mother?’ Can you imagine? So, you ride to that hospital, people do their thing, go back to the site and take another patient. But afterwards, you think about it. Like, is that family reunited? Does that child have parents? Is it an orphan? Our people could only really move on when they were sure about what had happened. [ … ] At a certain moment – and you don’t think about it at the time, but we had to evacuate the medical post. So, people were asking: why? I was helping a victim. And so we said (at the debriefing): ‘Look, we took every threat serious at that moment. There was serious chance that there was car bomb.’ And then: oh. So, they knew how to frame that.” (Firefighter, #20)

The main issues discussed at these debriefings were on the actions of everyone and not on their emotions. However, in most of the occupational groups, especially among firefighters, it seemed there was more a reliance on their immediate colleagues.

“We went there, we saw things, but the annoying thing is, it’s the people who stayed behind, those are the ones with questions and those need confirmation of ‘Yeah, yeah, everything is okay with us.’ And the stress goes away then too. And so we were told ‘The moment you hit a wall, it’ll come, but you know, we’re here, here is our contact number.’ Three days later, we were back at work. And that morning like ‘And?’ First night not great. But now, yeah. And you take care of each other. And that works. I did not really need the official stress team there. You work in a tight team with six. And you do that in team. If that doesn’t work, you go beyond that.” (Firefighter, #19)

In most emergency responder debriefings, both indirectly and directly exposed people were put together. In general, most post-response debriefings took on a similar form: explaining what happened, who did what, and hearing stories about what happened. These debriefings were mostly *ad hoc*, except with the firefighters, where it was part of their system.

“The evening itself, around 7, we had a debriefing. So, like, from X to our place. We told everyone. I personally found it really important to inform those who weren’t there to come. Else you get these two groups of those who were there and those who weren’t. That evening, almost everyone was there and we just let them talk. Mainly the people who were really there, of course. And a lot of the same stuff was told. And at night, I got a telephone call of someone saying it was good for them. And that they wanted to do it again, because they couldn’t sleep. Two days later, we did it again. And again, everyone does their story. So, general sketch, and then the story of what each of them had done. There were a lot of interesting stories. Really interesting. And people who were saying they were just broken physically. As if they had ran a marathon. And so, one said: ‘I’m glad I’m hearing this, because I thought I was the only one.’” (Red Cross volunteer, #11)

The only group where the systematic debriefings were not present seems to have been the police force. There were a couple of reasons for this. First, aid seemed to have been in a sort of one-on-one basis or on voluntary group basis. Second, as police had to provide security at the airport, both during the day and at night, they were relieved by the night shift. The shift that had experienced the attacks then directly went home, without knowing of debriefings. Third, they felt that those who provided aid were too inexperienced and young.

“We were … Look, there were people there. The stress team of the federal police was there. But … Look, this was extreme and Belgium did not have that expertise. The stress team was there and available. But, I had one session with them and they were like ‘Oh yeah, awful, isn’t it?’” (Police, #2)

## Discussion

In the current study, we looked at the different stressors and the psychological impact that emergency responders experienced during their response to the attacks on the national airport in Belgium. Our study shows the multitude of stressors that emergency responders faced and which all add to the extreme stress already experienced.

First, there was frustration with the lack of preparation and training. Although, in a sense, no one can properly prepare for the psychological impact of a terrorist attack (9), studies have shown that, when everything that can be prepared is in order, it might aid in reducing the “feeling” of not being prepared (23).

A major aspect of this was the inability to give tourniquets to victims who were bleeding out, which only military personnel were trained in. This, of course, caused a sense of helplessness because caregivers could not provide the care that they would want to provide nor they were able to provide psychological support to a victim because due to the circumstances there was no time.

The current study also shows that there are ethical decisions that emergency responders had to make: on whom are resources spend to save their lives? If there is a possible shooter, does one leave their patient to die? If one can tell family that their loved one has died, should one do so? However, these are ethical questions that emergency responders had no preparation for, no training, but had to decide on the spot.

A main frustration for indirectly exposed people was they were overprepared and had actually wanted more patients. Because of the disaster medicine system, they had provided fewer resources to one patient, who then had died, whereas, in normal circumstances, this person would have received full care. It is an indication that the transfer of patients to as many diverse hospitals as possible was a great success; thus, from an overarching organizational viewpoint, the lack of an overload of patients is positive. However, for the nurses, having to save resources in what they called “the calmest day ever” was frustrating.

All these abovementioned issues, such as “Who to save?,” are ethical conflicts. As found in other studies on terrorist attacks, not being able to provide proper care can cause severe stress and guilt for the responders (9, 24). This form of guilt related to what one should do but cannot do is called moral injury (25). Emergency responders should be better prepared for these ethical dilemmas, and, after a disaster, more attention should be provided for these moral injuries.

Second, the importance of leadership and teamwork was shown in the study as a protective factor. Proper leadership is important to provide not only organization and direction for emergency responders but also psychological support (26, 27). Although there was critique on the leadership and although it caused extra stress, the leadership did not seem to have been an issue for the adequacy of the emergency response. A reason for this is that emergency responders were still able to work together, because 1) they knew what their task was and, 2) by knowing other people, they were sure that the task would be completed properly. In that sense, trainings and simulations of disasters serve two main functions: 1) knowing how to respond to disasters and 2) knowing who will respond to disasters. As other researchers have noted, trainings and simulation exercises are probably why the response to other disasters in Europe occurred without too many issues (28, 29).

By knowing each other, it was also possible to improvise better. For example, communication issues such as the ones noted in our study are quite common during disasters (30). Indeed, they often put extra stress on emergency responders, as they are blinded in a certain way. However, through knowing the people on the field, social media was easily used to communicate with other people on the field. There was no need to exchange names or phone numbers, as these were already known.

Knowing what other colleagues were capable of provided a certain sense of security and comfort for emergency responders, as they were able to focus on their own tasks, instead of micromanaging (10). Such teamwork might act as a protective factor against the severe stress experienced during the acute phase (9, 31). However, police officers were often not present at such trainings, making cooperations at the airport more difficult.

The role of debriefings was also highlighted in the current study. The debriefings of these organizations followed quite closely international guidelines: they focused on the circumstances of the day, the functioning of the team, etc. (16). For a lot of these emergency responders, knowing who did what, what happened overall, and what happened to some of the people they took care of was enough. It would seem, therefore, that these debriefings were in no way “psychological debriefings,” which try to achieve emotional catharsis among emergency responders and have been found to quite often linked with either no improvement or an increase of PTSD (9, 32–34).

For police officers, there was an attempt for debriefing, but it seemed that it was mainly voluntarily, and, in fact, not all police officers knew that there was such an offer. Thus, several police officers went home in a daze. This might be because the airport police, railway police and the federal police located at the airport does not experience a lot of severe traumatic experiences. Hence, although there is a stress team at the police force, it seemed that how it was trained and put into action did not have the same effectiveness that other debriefings had. It might also explain why so many police officers sought out professional mental health aid later on.

The stark difference in the current study between, on the one hand, police officers and, on the other hand, all other emergency occupations might lie in the simple fact that police is generally not aimed at providing care. In other words, their role in emergencies is security. It might be that their structure of operations is, therefore, completely different from other emergency occupations, with other forms of debriefings and strict hierarchy. Furthermore, the role of police in such emergencies is normally to provide security for nurses and firefighters. However, in the current study, soldiers were present and thus better equipped to handle possible threats. In fact, soldiers were also better equipped to handle the injuries of most wounded. In addition, it seems that the police force does not feel that their role in large disasters was important enough, considering that they were noted to be missing from trainings. In other words, it seems that police officers do not seem to be prepared for large-scale disasters. This lack of direction and lack of specific tasks in their response to the attacks might also have contributed to difficulties to do debriefings such as those for other emergency responders. The reason being that it would have been difficult to go through the different tasks that everyone had to do as these were unclear from the start.

Another explanation might come from a social support perspective. Social support refers in most studies to the “perceived” social support, as in how much does a person think another person will support them when they are in need of aid (35). In general, the more such social support, the less likely a person will develop PTSD (36–38). As most emergency responders have trainings together, they might have a better idea who they can count on in the aftermath of occupational stressors. Studies do indeed show that emergency responders tend to feel their colleagues are the ones who understand what they went through and that responders who perceive a high social support will also have a lower chance of developing PTSD or major depression disorder (39–41). Certainly, the “John Wayne syndrome” of emergency responders might prohibit the support at times among emergency responders as well, but based on other studies, it seems that experiencing trainings together, being able to count on each other, can create the feeling of social connectedness (10, 41). Meaning that, although it might be difficult for emergency responders to discuss their feelings, they still have the feeling they can count on their colleagues. It might be that, for police officers, there was no way of finding social support among their colleagues because there were no trainings, no coordination, et cetera.

This would also explain why, among police officers, the majority of responders sought out professional mental health aid. The Conservation of Resources theory of stress states that, when resource loss circumstances are high, other resources such as social support become more important (42). As noted in other studies, emergency responders fit this theory. Emergency responders who face many occupational stressors but cannot rely on their social network or support might be more at risk of developing mental health issues (40). Thus, it might be that soldiers or nurses, who often have to work together and who do trainings together, have more a feeling that they can count on each other (thus, can rely on more resources) than police officers.

Our first recommendation is to ensure guidelines for debriefings for emergency responders. Although, certainly, occupations such as the firefighters are faced with enough disasters to have learnt from experience in how to organize debriefings, this was certainly not the case for police officers. Furthermore, it seems that the role of such debriefings should aim to be a psychological consult, but to provide answers to the questions that emergency responders might have. In addition, debriefings should not be viewed as something that is ready-made by the guidelines alone. In fact, who provides the debriefings plays a huge role. Firefighters are aided by colleagues, because these colleagues knew what responding to disasters is like, whereas police officers were debriefed by young psychologists who were seemingly detached from actual police work.

An important addition to this is that family of emergency responders could be better prepared on what to do after such a disaster. For example, to break down instances of the “John Wayne syndrome,” some authors have suggested that family and close friends of emergency responders can be taught psychological first aid (43). The main goal is then not to make friends and family the “therapist” of the emergency responders but to simply break down certain barriers in social support and to actually increase their social support (43). Furthermore, it is, in general, quite important to allow early interventions. Although psychological debriefings are not recommended, which generally occur within 24 h to 72 h after a traumatic event, other forms of early intervention have been shown to possible have an effect within the first three months after a traumatic event (44–46).

Second, terrorist attacks are uncommon but are not completely unpredictable. In fact, this was a period when, in neighboring countries, there were several attacks over the years (47). For example, the attack of 22 March 2016 happened around 4 months after the major attack in Paris of 13 November 2015, which was executed by terrorists from Brussels. Considering the current climate in Europe, with war in Ukraine, war in Gaza, and the very recent lone wolf attack in Brussels, emergency responders should be training for terrorist attacks, including stocking up on tourniquets, improving communications, and refreshing the principles of emergency medicine. Not only does this aid victims of such attacks but also those responding to the disasters, as they feel more secure and more able to do their tasks (48).

Third, considering such attacks and the disasters plans, emergency responder organizations should take into account the severe stress that is put on their personnel during such an attack. In that sense, disaster planning should never just include the response itself but also the possibility of personnel falling out in the aftermath of such attacks (28). In that sense, proper preparation for attacks also plays a role, as proper preparation will reduce the extreme stress experienced by the responders.

Fourth, the emotional stress that *ad hoc* ethical decisions pose should be taking into account in education and trainings or through some form of framework on how such decisions can be made. Studies indicate that responders during disasters often not only face such ethical choices but also are left to their own coping skills (24). Increasingly, such ethical considerations become part of disaster protocol plans (49). However, it is important that such considerations are not only provided in plans but that health care providers are early on trained to make and handle such decisions. It is clear that there needs to be not only more research but also more training on how ethical frameworks during disasters (50).

This study is limited by several factors. First, considering this study was performed 2 years after the attacks, there will be possible recall bias. Second, the gender balance was very uneven, as well as the age balance. It might affect the results as studies suggest that younger people and women experience more psychological stress from terrorist attacks (2, 7, 51, 52). However, occupations such as firefighters, soldiers, and police are done more often by men than women. Third, our study focused on the first 24 h. Naturally, the work pressure on hospitals endures beyond the 24 h mainly described in this study, because of the treatment of severely wounded people, in an atmosphere of threat (29). Thus, the stressors and pressure that were put on indirectly exposed hospital staff might be larger in the days afterward.

Nonetheless, the current study is one of the few studies to focus on the many different forms of stressors that emergency responders can experience throughout terrorist attacks. Furthermore, few studies have looked at what post-response debriefings consist of. Considering the difficulty in the field to differentiate between psychological debriefings and other types of debriefings, this seems especially worthwhile (9). The relatively large dataset also adds to the strength of the study. Finally, for public health interventions and therapists, the insights of this study are invaluable, as very few studies try to provide insight into what emergency responders go through during their action, which is important, as victims, in general, have noted that they have the feeling of not being understood by therapists or their social support (17). Further studies should try to differentiate even more between the different stressors and post-action response, especially in the long term. More studies should also try to focus on how debriefings are organized, as it remains difficult to differentiate between psychological debriefings and other types while also showing the positive effect (or lack of effect) of such debriefings for mental health (9, 31, 53, 54). Finally, studies on the ethical issues that emergency responders encounter might also be helpful, as such ethical issues also weigh on emergency responders.

## Data availability statement

The datasets presented in this article are not readily available because this is qualitative data on sensitive subjects. Requests to access the datasets should be directed to roel.van.overmeire@vub.be.

## Ethics statement

The studies involving humans were approved by ethics commission of the UZ Brussels/VUB. The studies were conducted in accordance with the local legislation and institutional requirements. The participants provided their written informed consent to participate in this study. Written informed consent was obtained from the individual(s) for the publication of any potentially identifiable images or data included in this article.

## Author contributions

EM: Conceptualization, Data curation, Formal analysis, Investigation, Methodology, Project administration, Software, Writing – original draft, Writing – review & editing. LV: Data curation, Formal analysis, Funding acquisition, Methodology, Project administration, Supervision, Validation, Writing – review & editing. HV: Funding acquisition, Investigation, Project administration, Software, Supervision, Validation, Writing – review & editing. SV: Methodology, Writing – review & editing. JB: Formal analysis, Funding acquisition, Project administration, Resources, Supervision, Visualization, Writing – review & editing. RV: Conceptualization, Data curation, Formal analysis, Investigation, Methodology, Software, Supervision, Writing – original draft, Writing – review & editing.

## References

[1] Skogstad L Heir T Hauff E Ekeberg Ø. Post-traumatic stress among rescue workers after terror attacks in Norway. Occup Med (Chic Ill) (2016) 66:528–35. doi: 10.1093/occmed/kqw063 27325417

[2] Perlman SE Friedman S Galea S Nair HP Erős-Sarnyai M Stellman SD. Short-term and medium-term health effects of 9/11. Lancet (2011) 378:925–34. doi: 10.1016/S0140-6736(11)60967-7 21890057

[3] Gjerland A Pedersen MJB Ekeberg Ø Skogstad L. Sick-leave and help seeking among rescue workers after the terror attacks in Norway, 2011. Int J Emerg Med (2015) 8:31. doi: 10.1186/s12245-015-0081-4 26283071 PMC4539308

[4] De Stefano C Orri M Agostinucci JM Zouaghi H Lapostolle F Baubet T. Early psychological impact of Paris terrorist attacks on healthcare emergency staff: A cross-sectional study. Depress Anxiety (2018) 35:275–82. doi: 10.1002/da.22724 29421842

[5] Meudal J Vandentorren S Simeoni L Denis C. French red cross volunteer rescue workers. J Nervous Ment Dis (2020) 208:413–7. doi: 10.1097/NMD.0000000000001143 31985563

[6] Wesemann U Zimmermann P Mahnke M Butler O Polk S Willmund G. Burdens on emergency responders after a terrorist attack in Berlin. Occup Med (Chic Ill) (2018) 68:60–3. doi: 10.1093/occmed/kqx172 29309698

[7] Wesemann U Helms C Polk S Mahnke M Bühler A Muschner P. Mistrust among rescue workers after the terrorist attack in berlin in 2016 – gender-specific health inequality. Disaster Med Public Health Prep (2023) 17:e394. doi: 10.1017/dmp.2023.77 37183713

[8] Wesemann U Bühler A Mahnke M Polk S Willmund G. Longitudinal mental health effects of the 2016 terrorist attack in berlin on various occupational groups of emergency service personnel. Health Secur (2020) 18:403–8. doi: 10.1089/hs.2019.0108 33616414

[9] Skryabina EA Betts N Amlôt R Reedy G. Understanding the psychological impacts of responding to a terrorist incident. Eur J Psychotraumatol (2021) 12. doi: 10.1080/20008198.2021.1959116 PMC8635676 34868476

[10] Van Puyvelde M Van Herck J Van den Bossche J Goethals F Gijbels D Detaille F. Walk the line: a systemic perspective on stress experienced by emergency medical personnel by comparing military and civilian prehospital settings. Front Public Health (2023) 11:1136090. doi: 10.3389/fpubh.2023.1136090 37441639 PMC10335750

[11] Wesemann U Applewhite B Himmerich H. Investigating the impact of terrorist attacks on the mental health of emergency responders: systematic review. BJPsych Open (2022) 8:e107. doi: 10.1192/bjo.2022.69 35656574 PMC9230690

[12] Durodié B Wainwright D. Terrorism and post-traumatic stress disorder: a historical review. Lancet Psychiatry (2019) 6:61–71. doi: 10.1016/S2215-0366(18)30335-3 30342864 PMC9939936

[13] Smith EC Holmes L Burkle FM. The physical and mental health challenges experienced by 9/11 first responders and recovery workers: A review of the literature. Prehosp Disaster Med (2019) 34:625–31. doi: 10.1017/S1049023X19004989 31625489

[14] Stene LE Vuillermoz C Van Overmeire R Bilsen J Dückers M Nilsen LG. Psychosocial care responses to terrorist attacks: a country case study of Norway, France and Belgium. BMC Health Serv Res (2022) 22:390. doi: 10.1186/s12913-022-07691-2 35331222 PMC8953389

[15] Bisson JI. Early responding to traumatic events. Br J Psychiatry (2014) 204:329–30. doi: 10.1192/bjp.bp.113.136077 24785764

[16] Knobler HY Nachshoni T Jaffe E Peretz G Yehuda Ben Y. Psychological guidelines for a medical team debriefing after a stressful event. Mil Med (2007) 172:581–5. doi: 10.7205/MILMED.172.6.581 17615836

[17] Van Overmeire R Muysewinkel E Van Keer R-L Vesentini L Bilsen J. Victims of the terrorist attacks in Belgium and professional mental health aid barriers: A qualitative study. Front Psychiatry (2021) 12:638272. doi: 10.3389/fpsyt.2021.638272 34276430 PMC8283008

[18] McNally RJ. Remembering Trauma. Cambridge, Massachusetts: Belknap Press/Harvard University Press (2003).

[19] Galea S Ahern J Resnick H Kilpatrick D Bucuvalas M Gold J. Psychological sequelae of the september 11 terrorist attacks in New York City. New Engl J Med (2002) 346:982–7. doi: 10.1056/NEJMsa013404 11919308

[20] Willig C. Interpretation and analysis. In: Flick U, editor. The Sage Handbook of Qualitative Data Analysis. London, UK: Sage (2014). doi: 10.4135/9781446282243

[21] Walsh RS McClean B Doyle N Ryan S Scarborough-Lang S-J Rishton A. A thematic analysis investigating the impact of positive behavioral support training on the lives of service providers: “It makes you think differently”. Front Psychol (2019) 10:2408. doi: 10.3389/fpsyg.2019.02408 31736821 PMC6828942

[22] Fielden Amy L Sillence E Little L. Children’s understandings’ of obesity, a thematic analysis. Int J Qual Stud Health Well-being (2011) 6:7170. doi: 10.3402/qhw.v6i3.7170 PMC3166520 21897830

[23] Skryabina EA Betts N Reedy G Riley P Amlôt R. The role of emergency preparedness exercises in the response to a mass casualty terrorist incident: A mixed methods study. Int J Disaster Risk Reduction (2020) 46:101503. doi: 10.1016/j.ijdrr.2020.101503 PMC7709486 33312855

[24] Gustavsson ME Juth N Arnberg FK. Dealing with difficult choices: a qualitative study of experiences and consequences of moral challenges among disaster healthcare responders. Confl Health (2022) 16:24. doi: 10.1186/s13031-022-00456-y 35527276 PMC9079207

[25] D’Alessandro AM Ritchie K McCabe RE. Healthcare workers and COVID-19-related moral injury: an interpersonally-focused approach informed by PTSD. Front Psychiatry (2022) 12:784523. doi: 10.3389/fpsyt.2021.784523 35264983 PMC8900218

[26] Kowalski C. Leadership of first-responders following trauma. J Bus Contin Emer Plan (2019) 13(1):81–90. 31462365

[27] Persoff J Ornoff D Little C. The role of hospital medicine in emergency preparedness: A framework for hospitalist leadership in disaster preparedness, response, and recovery. J Hosp Med (2018) 13:713–8. doi: 10.12788/jhm.3073 30261086

[28] Moran CG Webb C Brohi K Smith M Willett K. Lessons in planning from mass casualty events in UK. BMJ (2017) 359:j4765. doi: 10.1136/bmj.j4765 29070605

[29] Hirsch M Carli P Nizard R Riou B Baroudjian B Baubet T. The medical response to multisite terrorist attacks in Paris. Lancet (2015) 386:2535–8. doi: 10.1016/S0140-6736(15)01063-6 26628327

[30] Goralnick E Halpern P Loo S. Leadership during the Boston marathon bombings: A qualitative after-action review. Disaster Med Public Health Prep (2015) 9:489–95. doi: 10.1017/dmp.2015.42 26094685

[31] Gouweloos-Trines J Tyler MP Giummarra MJ. Perceived support at work after critical incidents and its relation to psychological distress: a survey among prehospital providers. Emergency Med J (2017) 34:816–22. doi: 10.1136/emermed-2017-206584 29055892

[32] Tran DV North CS. The association between dissatisfaction with debriefing and post-traumatic stress disorder (PTSD) in rescue and recovery workers for the Oklahoma City bombing. Disaster Med Public Health Prep (2018) 12:718–22. doi: 10.1017/dmp.2017.153 29540249

[33] Van Overmeire R. The myth of psychological debriefings during the corona pandemic. J Glob Health (2020) 10. doi: 10.7189/jogh.10.020344 PMC7689189 33274056

[34] Eppich WJ Mullan PC Brett-Fleegler M. “Let’s talk about it”: translating lessons from health care simulation to clinical event debriefings and coaching conversations. Clin Pediatr Emerg Med (2016) 17:200–11. doi: 10.1016/j.cpem.2016.07.001

[35] Thoresen S Jensen TK Wentzel-Larsen T. Social support barriers and mental health in terrorist attack survivors. J Affect Disord (2014) 156:187–93. doi: 10.1016/j.jad.2013.12.014 24398044

[36] Wang Y Chung MC Wang N. Social support and posttraumatic stress disorder: A meta-analysis of longitudinal studies. Clin Psychol Rev (2021) 85:101998. doi: 10.1016/j.cpr.2021.101998 33714168

[37] Kaniasty K Norris FH. Longitudinal linkages between perceived social support and posttraumatic stress symptoms: Sequential roles of social causation and social selection. J Trauma Stress (2008) 21:274–81. doi: 10.1002/jts.20334 18553415

[38] Clapp JD Gayle Beck J. Understanding the relationship between PTSD and social support: The role of negative network orientation. Behav Res Ther (2009) 47:237–44. doi: 10.1016/j.brat.2008.12.006 PMC2656396 19162260

[39] Prati G Pietrantoni L. The relation of perceived and received social support to mental health among first responders: a meta-analytic review. J Community Psychol (2010) 38:403–17. doi: 10.1002/jcop.20371

[40] Kshtriya S Kobezak HM Popok P. Social support as a mediator of occupational stressors and mental health outcomes in first responders. J Community Psychol (2020) 48:2252–63. doi: 10.1002/jcop.22403 32841385

[41] Van Overmeire R Van Keer R Bilsen J. Impact of terrorist attacks on social relationships. Clin Psychol Psychother (2021) 28:1472–81. doi: 10.1002/cpp.2587 33768615

[42] Hobfoll SE Halbesleben J Neveu JP Westman M. Conservation of resources in the organizational context: The reality of resources and their consequences. Annu Rev Organizational Psychol Organizational Behav (2018) 5:103–28. doi: 10.1146/annurev-orgpsych-032117-104640

[43] O’Toole M Mulhall C Eppich W. Breaking down barriers to help-seeking: preparing first responders’ families for psychological first aid. Eur J Psychotraumatol (2022) 13. doi: 10.1080/20008198.2022.2065430 PMC9103391 35572389

[44] Roberts NP Kitchiner NJ Kenardy J. Systematic review and meta-analysis of multiple-session early interventions following traumatic events. Am J Psychiatry (2009) 166:293–301. doi: 10.1176/appi.ajp.2008.08040590 19188285

[45] Roberts NP Kitchiner NJ Kenardy J. Multiple session early psychological interventions for the prevention of post-traumatic stress disorder. Cochrane Database Systematic Rev (2009) (3):CD006869. doi: 10.1002/14651858.CD006869.pub2 19588408

[46] Roberts NP Kitchiner NJ Kenardy J Bisson JI. Early psychological interventions to treat acute traumatic stress symptoms. Cochrane Database Systematic Rev (2010) (3):CD007944. doi: 10.1002/14651858.CD007944.pub2 PMC11491193 20238359

[47] European Council. Infographic - Terrorism in the EU: facts and figures. Available at: https://www.consilium.europa.eu/en/infographics/terrorism-eu-facts-figures/.

[48] FOD Volksgezondheid veiligheid van de voedselketen en milieu. Technische werkgroep: Psychosociale opvolging: voor een geïntegreerde psychosociale opvolging van getroffenen van collectieve noodsituaties. Brussels, Belgium: Federal government (2018).

[49] Leider JP DeBruin D Reynolds N Koch A Seaberg J. Ethical guidance for disaster response, specifically around crisis standards of care: A systematic review. Am J Public Health (2017) 107:e1–9. doi: 10.2105/AJPH.2017.303882 PMC5551597 28727521

[50] Cuthbertson J Penney G. Ethical decision making in disaster and emergency management: A systematic review of the literature. Prehosp Disaster Med (2023) 38:622–7. doi: 10.1017/S1049023X23006325 PMC10548018 37675490

[51] Darves-Bornoz J-M Alonso J de Girolamo G de Graaf R Haro J-M Kovess-Masfety V. Main traumatic events in Europe: PTSD in the European study of the epidemiology of mental disorders survey. J Trauma Stress (2008) 21(5):455–62. doi: 10.1002/jts.20357 18956444

[52] Wesemann U Mahnke M Polk S. Long-term effects of the terror attack in Berlin in 2016 on paranoid ideation in female emergency personnel. BJPsych Open (2020) 6:e79. doi: 10.1192/bjo.2020.57 32741399 PMC7453799

[53] Brooks SK Dunn R Amlôt R. Social and occupational factors associated with psychological distress and disorder among disaster responders: a systematic review. BMC Psychol (2016) 4:18. doi: 10.1186/s40359-016-0120-9 27114240 PMC4845476

[54] Brooks SK Dunn R Amlôt R. Training and post-disaster interventions for the psychological impacts on disaster-exposed employees: a systematic review. J Ment Health (2018), 1–25. doi: 10.1080/09638237.2018.1437610 29447058

